# A meta‐analysis of contrast‐enhanced spectral mammography versus MRI in the diagnosis of breast cancer

**DOI:** 10.1111/1759-7714.13400

**Published:** 2020-03-31

**Authors:** Wanqing Xiang, Haiying Rao, Liyu Zhou

**Affiliations:** ^1^ Department of Radiology Lishui People's Hospital Lishui China; ^2^ Department of Clinical Laboratory Lishui People's Hospital Lishui China

**Keywords:** Contrast‐enhanced spectral mammography, diagnosis breast cancer, meta‐analysis, MRI

## Abstract

**Background:**

To identify the performance of contrast‐enhanced spectral mammography (CESM) and magnetic resonance imaging (MRI) for breast cancer diagnosis by pooling the open published data.

**Methods:**

A systematic review of studies relevant to CESM and MRI in the diagnosis of breast cancer were screened in the electronic databases of Pubmed, EMBASE, the Cochrane Library, Web of Science, Google scholar and CNKI. The methodical quality of the included publications was evaluated by the quality assessment of diagnostic accuracy studies‐2 (QUADAS‐2). The diagnostic sensitivity, specificity and area under the ROC curve (AUC) were pooled and the true positive (TP), false positive (FP), false negative (FN) and true negative (TN) of the original studies were calculated.

**Results:**

A total of 13 diagnostic publications were identified and included in the meta‐analysis. Of those included, five were retrospective studies and the remaining eight were prospective work. The combined data indicating the pooled sensitivity and specificity of CESM and MRI were 0.97 (95% CI: 0.95–0.98), 0.66 (95% CI: 0.59–0.71), 0.97 (95% CI: 0.95–0.98),and 0.52 (95% CI: 0.46–0.58), respectively. The pooled +LR and –LR for CESM were 2.70 (95% CI: 1.57–4.65), 0.06 (95% CI: 0.04–0.09), and 2.01 (95% CI: 1.78–2.26), 0.08 (95% CI: 0.05–0.11) for MRI, respectively. For the diagnostic odds ratio (DOR), the pooled results of CESM and MRI were 60.15 (95% CI: 24.72–146.37) and 31.34 (95% CI: 19.61–50.08), respectively. The AUC of the symmetric receiver operating characteristic curve (SROC) was 0.9794 and 0.9157 for CESM and MRI, respectively, calculated using the Moses model in the diagnosis of breast cancer.

**Conclusions:**

Both CESM and MRI are effective methods for the detection of breast cancer with high diagnostic sensitivity. The diagnostic performance of CESM appears to be more effective than MRI.

## Introduction

Breast cancer is one of the most diagnosed malignant cancers in females worldwide.^1^, ^2^ Early diagnosis and proper treatment is key for the long‐term prognosis of breast cancer patients. Mammography is a common examination for breast cancer screening which has been in clinical use for a long period of time. Contrast‐enhanced spectral mammography (CESM), which utilizes dual energy for mammographic acquisition with intravenous iodine contrast agent administration, represents a relatively new diagnostic tool that was first approved by the FDA for breast cancer screening in 2011. According to the literature, the sensitivity of CESM has been reported to range from 93%–100%.^3^, ^4^ Magnetic resonance imaging (MRI) for the detection of breast lesions has also been proven to be effective with relatively high sensitivity. Several studies^5^, ^6^, ^7^ have previously compared the diagnostic performance of CESM and MRI in breast cancer screening. However, the conclusions of these studies were different because of the small sample size and study designs. In order to further evaluate the diagnostic performance of CESM and MRI in breast cancer identification, we performed this meta‐analysis to provide the strongest level of evidence on which to guide future decisions.

## Methods

### Screening of publications

A systematic review of publications relevant to CEMS and MRI in the diagnosis of breast cancer was undertaken using the electronic databases of Pubmed, EMBASE, the Cochrane Library, Web of Science, Google scholar and CNKI. The electronic search words were as follows: “breast cancer” or “breast carcinoma” or breast mass”; “CESM” or contrast‐enhanced spectral mammography” or “spectral contrast‐enhanced mammography” or “MRI” or “Magnetic Resonance Imaging”. The relevant studies identified in the databases were screened by two reviewers (W. Xiang & H. Rao) independently. The studies considered potentially suitable for the inclusion criteria are detailed in the main text. The references of the studies were also screened in order to identify those suitable for inclusion.

### Inclusion of studies and data extraction

After screening the relevant studies in the databases, all the included diagnostic trials were required to fulfill the following inclusion and exclusion criteria. Inclusion criteria were: (i) the study population were cases with a confirmed diagnosis of breast cancer; (ii) diagnostic methods used were CEMS or MRI; (iii) the gold diagnostic standard for breast cancer was pathological examination; (iv) the diagnostic parameter such as true positive (TP), false positive (FP), false negative (FN) and true negative (TN) could be extracted from the original studies. The publication exclusion criteria were: (i) case report or review study type; (ii) breast cancer had not been confirmed by pathological examination; (iii) duplicated publication or data.

### Data extraction

The general information and important data were extracted from the original studies by two reviewers independently and a cross check undertaken. The general information such as authors, year of paper publication, sample size, study type were extracted from each of the included studies. The data for calculating the diagnostic performance such as TP, FP, FN and TN were also carefully extracted from the original publications by the two reviewers independently.

### Quality assessment

The methodological quality of the included publications was evaluated by the quality assessment of diagnostic accuracy studies‐2 (QUADAS‐2). QUADAS‐2 mainly focuses on the following aspects: patient selection, index test reference standard and flow and timing, which present the main quality of the diagnostic study. If the study fulfills the above criteria, it is of low risk of bias, otherwise it is considered to be of high risk of bias.

### Statistical analysis

Statistical heterogeneity across the included 13 publications were firstly evaluated by the I^2^ test. The data was pooled by fixed effect model if there was no statistical heterogeneity. Otherwise, the data was pooled by random effect model. The diagnostic sensitivity and specificity were pooled according to the equation sensitivity = true positive/(true positive + false negative), specificity = true negative/(true negative + false positive). Two‐tailed *P* < 0.05 was deemed to be statistically significant.

## Results

### Details of included studies

**Figure 1:**
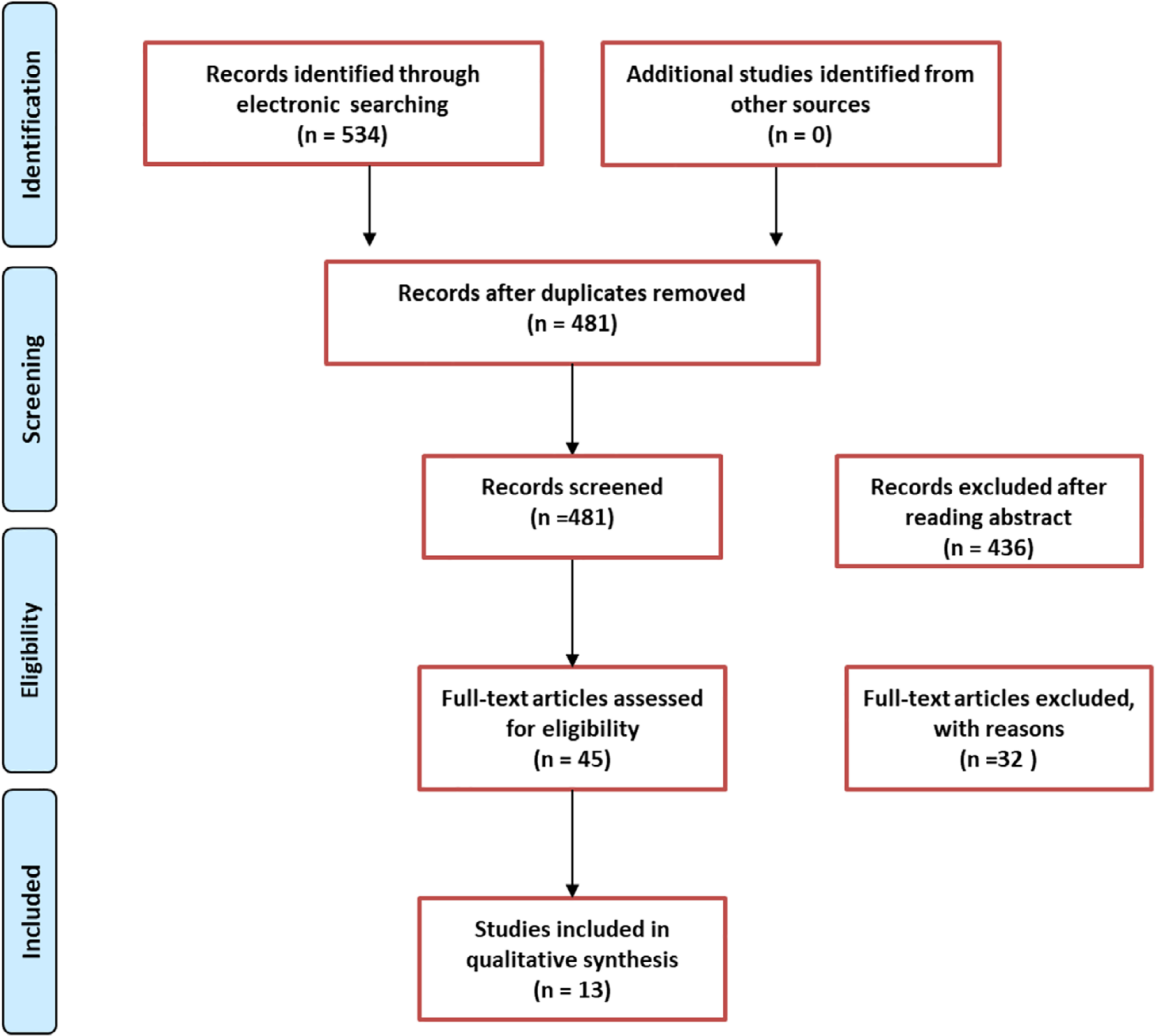
Study flowchart.

After a systematic search of the relevant electronic databases, 534 publications were initially identified, and after removal of duplicated data, studies and noncorrelated studies, 13 diagnostic publications^5^, ^6^, ^7^, ^8^, ^9^, ^10^, ^11^, ^12^, ^13^, ^14^, ^15^, ^16^, ^17^ were identified and included in the final meta‐analysis (Fig 1). Of those included, five were retrospective studies and the other eight were prospective work. The general characteristics of the 13 studies included in the meta‐analysis are demonstrated in Table 1.

**Table 1:** General characteristics of publications included in the meta‐analysis

						Lesions detected	
Study	Year	Age	Study design	Sample size	Lesions	CESM	MRI
Dromain *et al*.^23^	2011	NA	Prospective	42	37	35	37
Jochelson *et al*.^8^	2013	49 (median)	Prospective	52	52	50	50
Zhang *et al*.^9^	2014	53.3 ± 11.7	Prospective	63	63	63	63
Lucyńska *et al*.^6^	2015	NA	Retrospective	102	118	106	107
Luna *et al*.^5^	2015	NA	Retrospective	48	50	50	50
Wang *et al*.^10^	2016	52.9 ± 10.7	Prospective	68	77	77	77
Xu^16^	2017	46.8 ± 11.7	Prospective	40	53	53	53
Fallenberg *et al*.^12^	2017	52.8 ± 12.6	Prospective	155	155	155	155
Yu & Li^15^	2017	52.4 ± 2.4	Retrospective	30	36	34	34
Jiang *et al*.^14^	2017	50.0 ± 9.0	Retrospective	145	153	151	149
Li *et al*.^11^	2017	56.0 ± 10.6	Retrospective	48	66	64	66
Lee‐Felker *et al*.^13^	2017	50 (median)	Prospective	52	120	120	120
Zou *et al*.^17^	2018	48.1 ± 10.4	Prospective	73	91	85	87

### Quality of included studies

**Figure 2:**
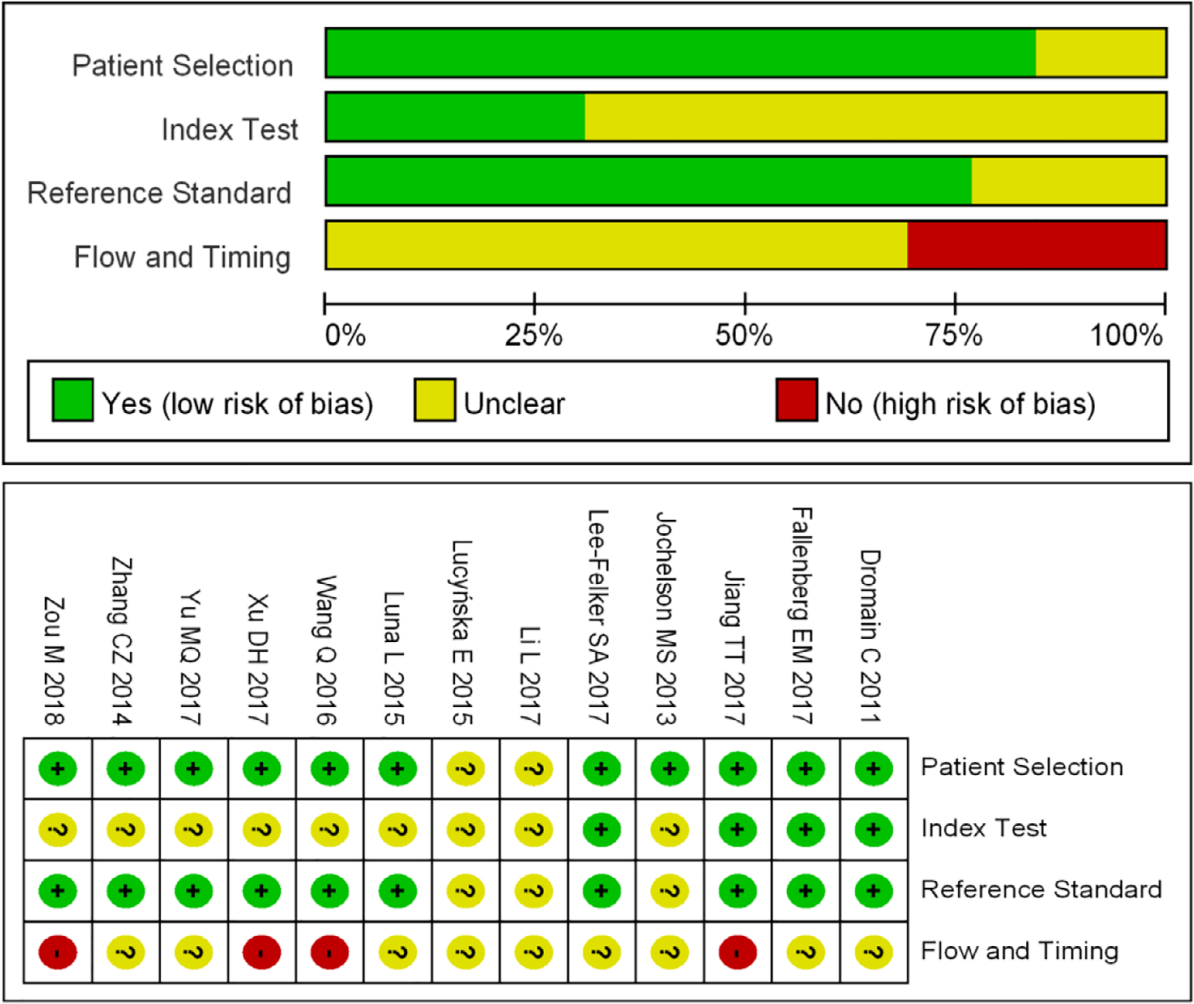
Quality evaluation of studies included in the meta‐analysis.

The methodical quality of the included publications was evaluated by QUADAS‐2 (Fig 2). Most of the studies (11) reported the selection criteria of the patients to be at low risk of bias in this respect. However, none of the 13 studies provided a conclusion with regard to the flow and timing. Therefore, the bias was at high risk, which may decrease the quality of the publications.

### Meta‐analysis

**Figure 3:**
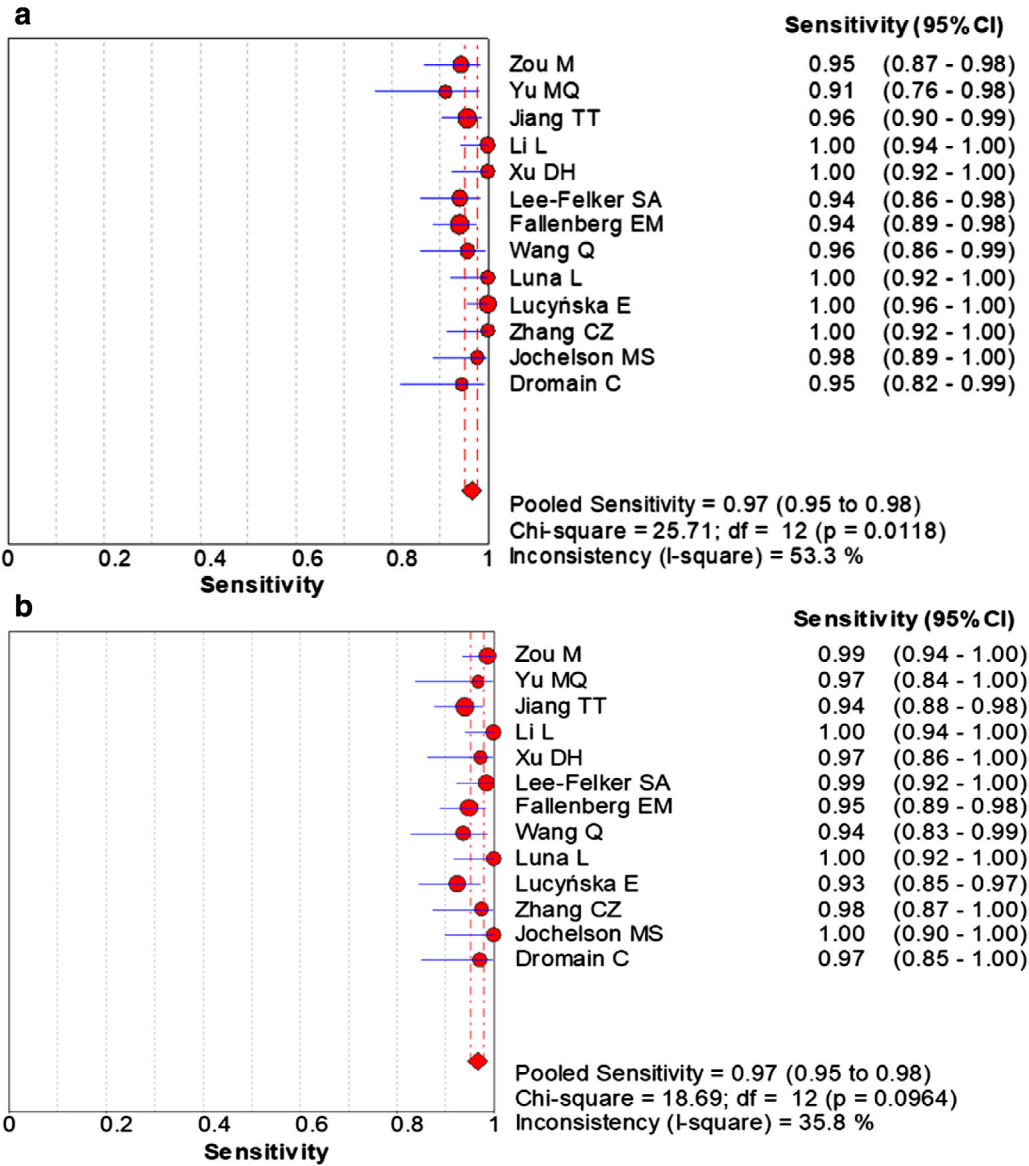
Forest plot of the pooled diagnostic sensitivity of CESM and MRI for breast cancer. (**a**) Forest plot of sensitivity of CESM in the diagnosis of breast cancer; (**b**) Forest plot of sensitivity of MRI in the diagnosis of breast cancer.

**Figure 4:**
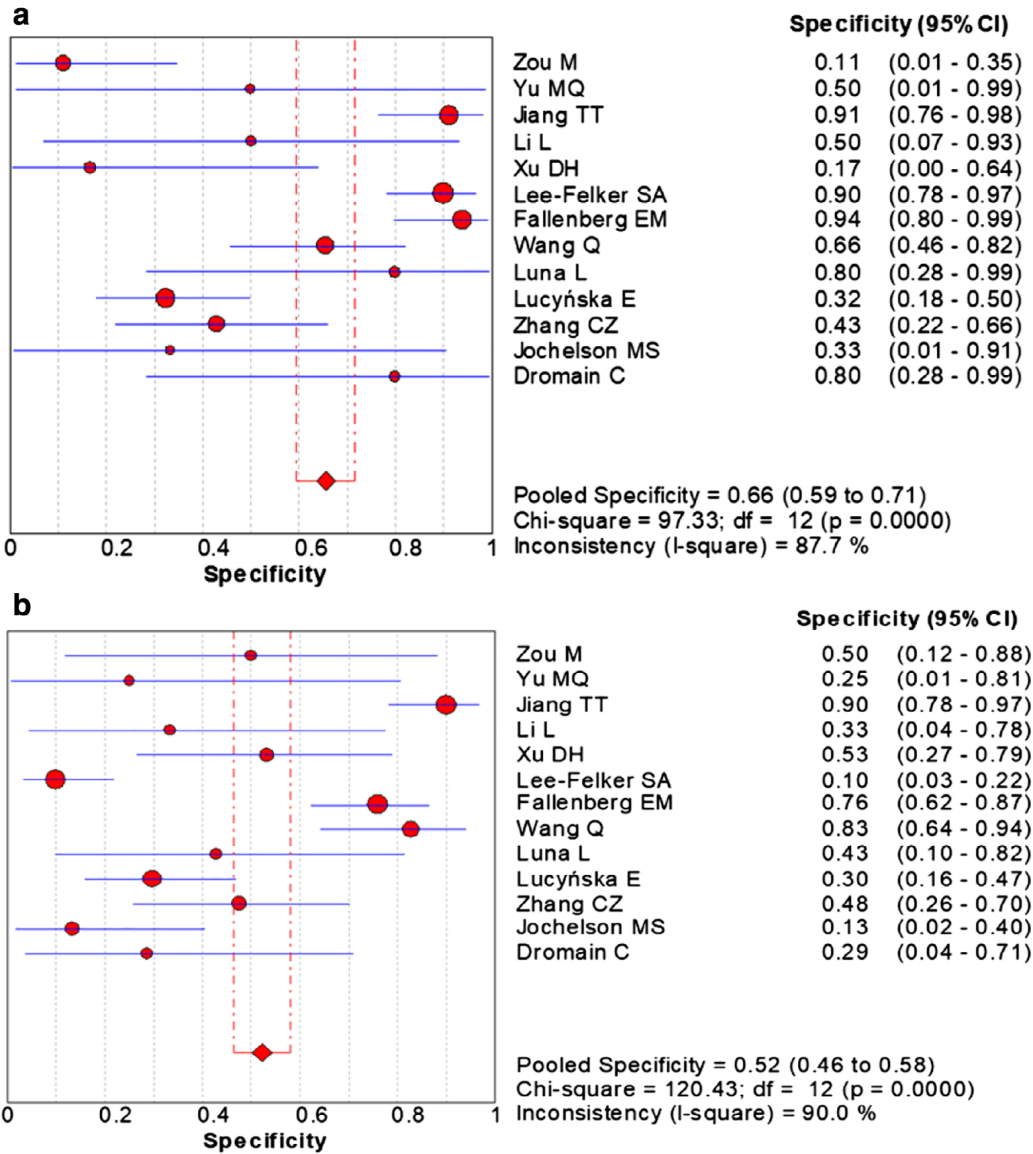
Forest plot of the pooled diagnostic specificity of CESM and MRI for breast cancer. (**a**) Forest plot of specificity of CESM in the diagnosis of breast cancer; (**b**) Forest plot of specificity of MRI in the diagnosis of breast cancer.

**Figure 5:**
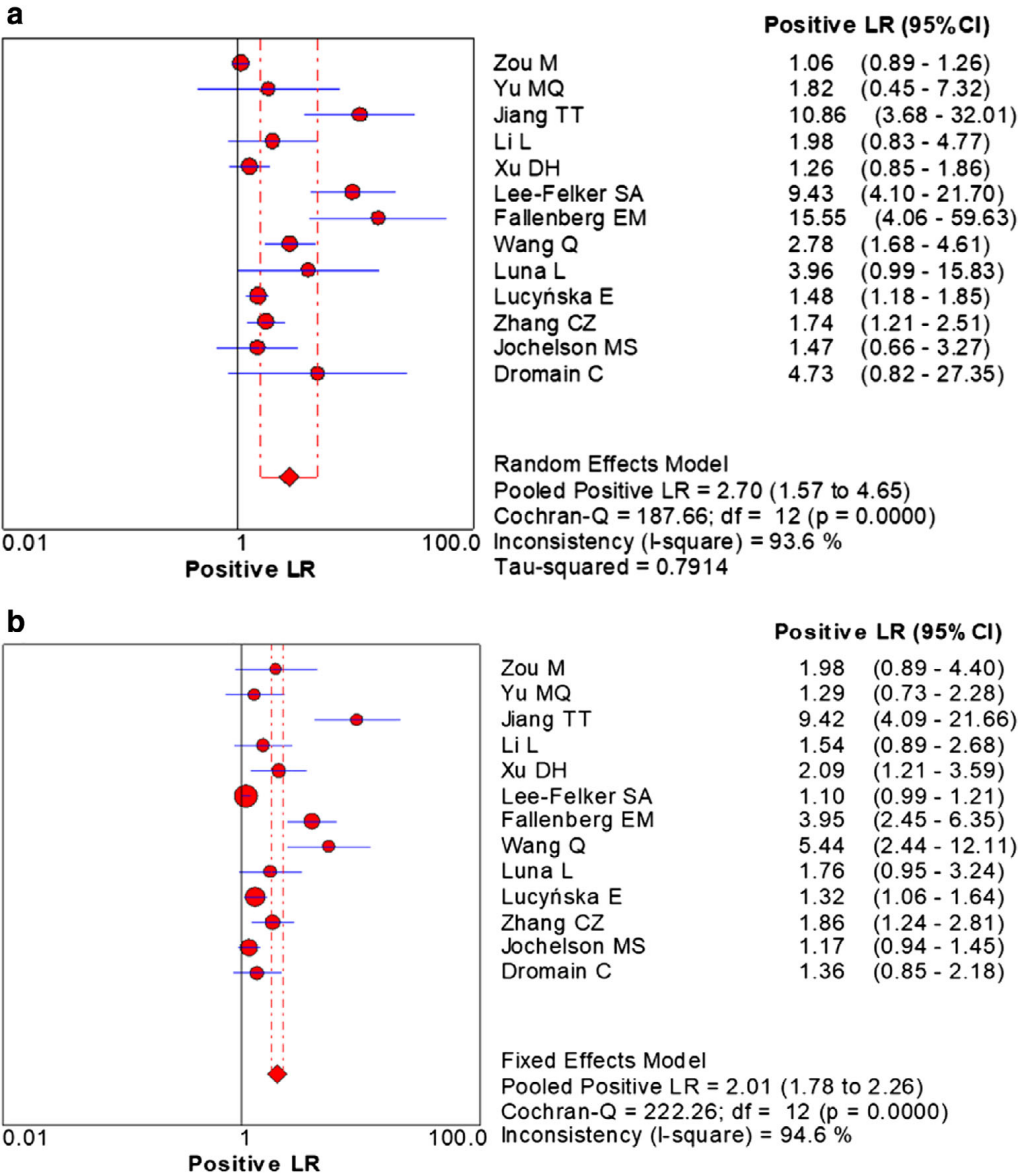
Forest plot of the pooled +LR of CESM and MRI for breast cancer. (**a**) Forest plot of +LR for CESM in the diagnosis of breast cancer; (**b**) Forest plot of +LR for MRI in the diagnosis of breast cancer.

**Figure 6:**
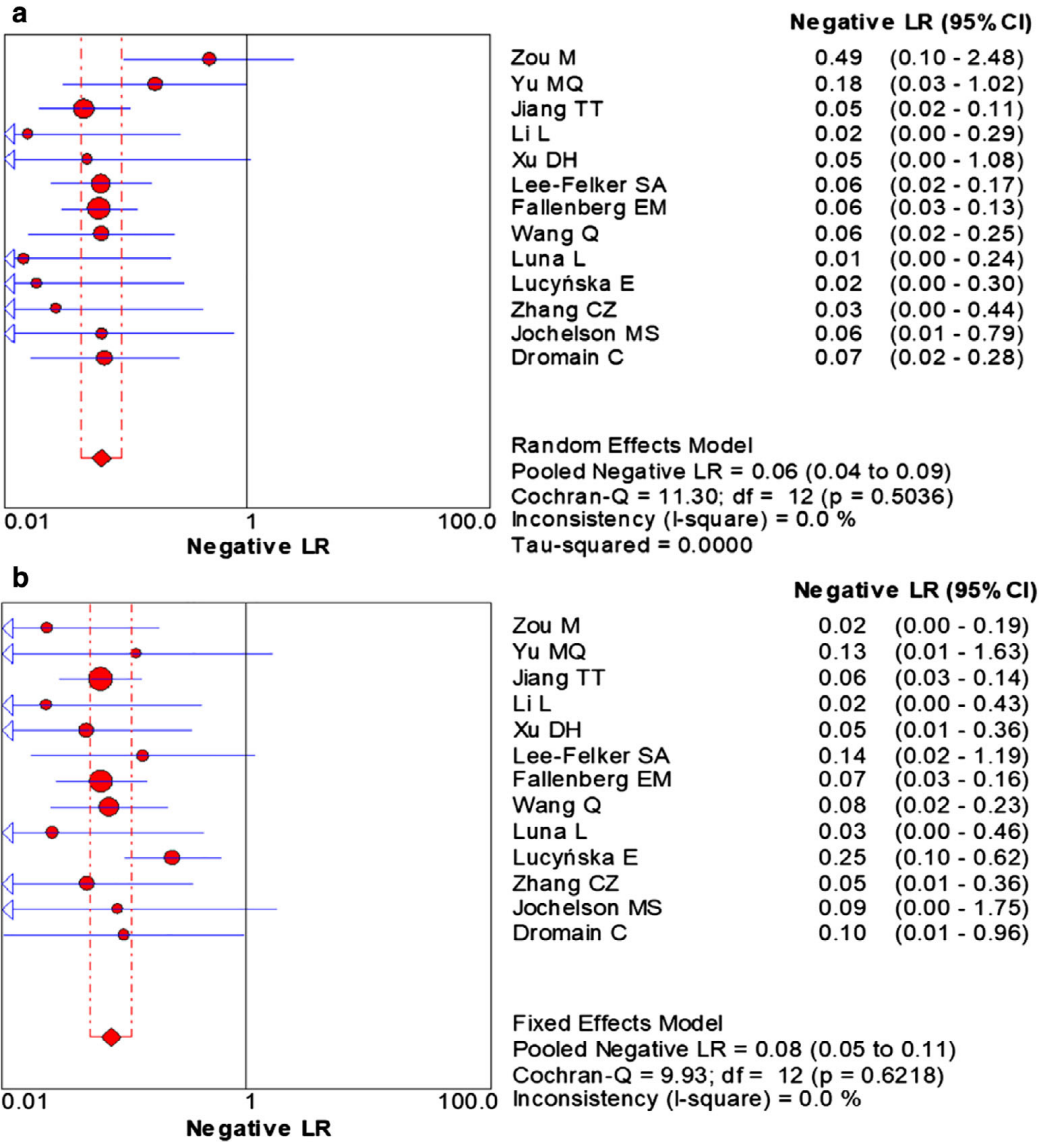
Forest plot of the pooled –LR of CESM and MRI for breast cancer. (**a**) Forest plot of –LR for CESM in the diagnosis of breast cancer; (**b**) Forest plot of –LR for MRI in the diagnosis of breast cancer.

**Figure 7:**
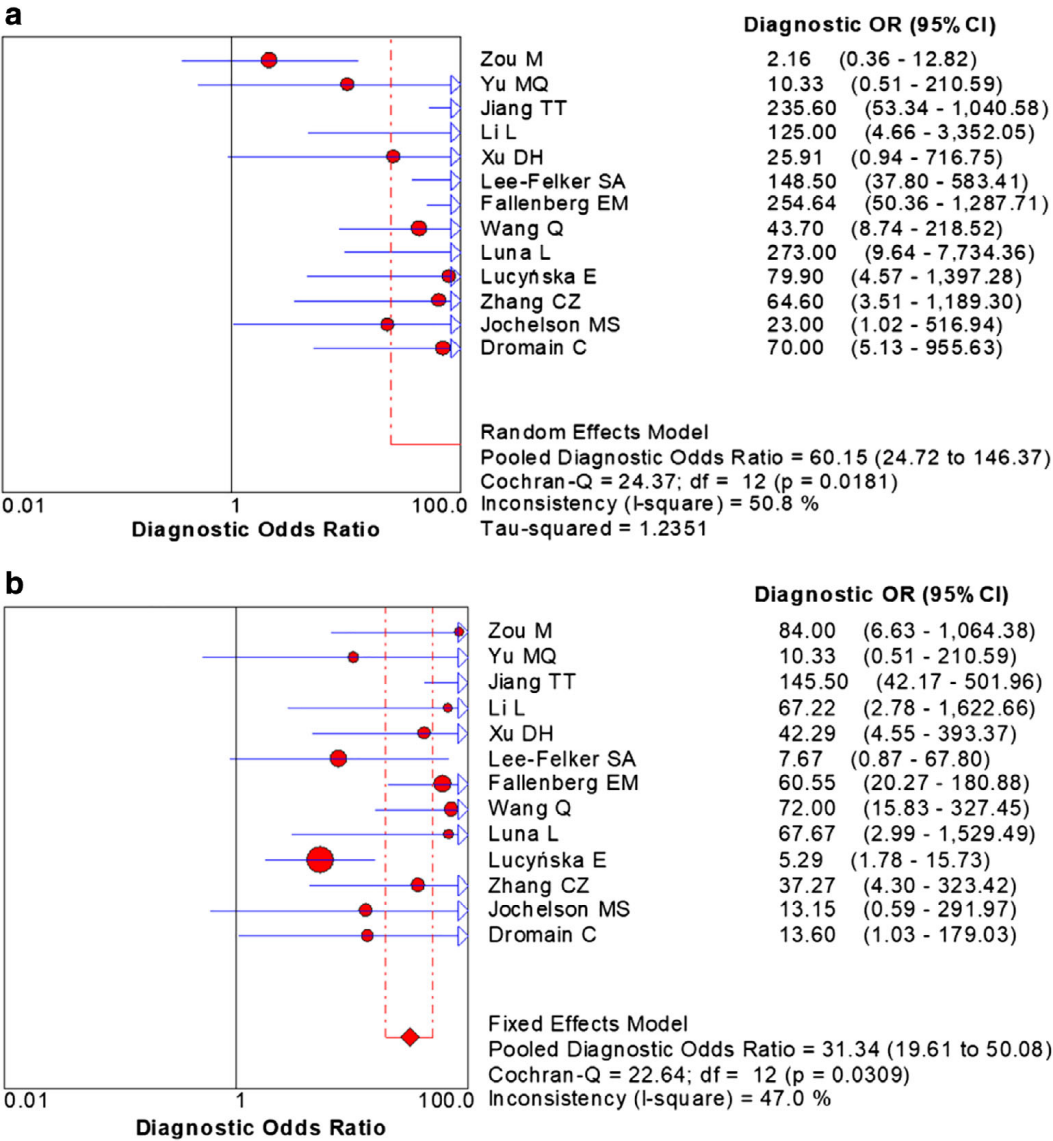
Forest plot of the pooled DOR of CESM and MRI for breast cancer. (**a**) Forest plot of DOR for CESM in the diagnosis of breast cancer; **(b**) Forest plot of DOR for MRI in the diagnosis of breast cancer.

**Figure 8:**
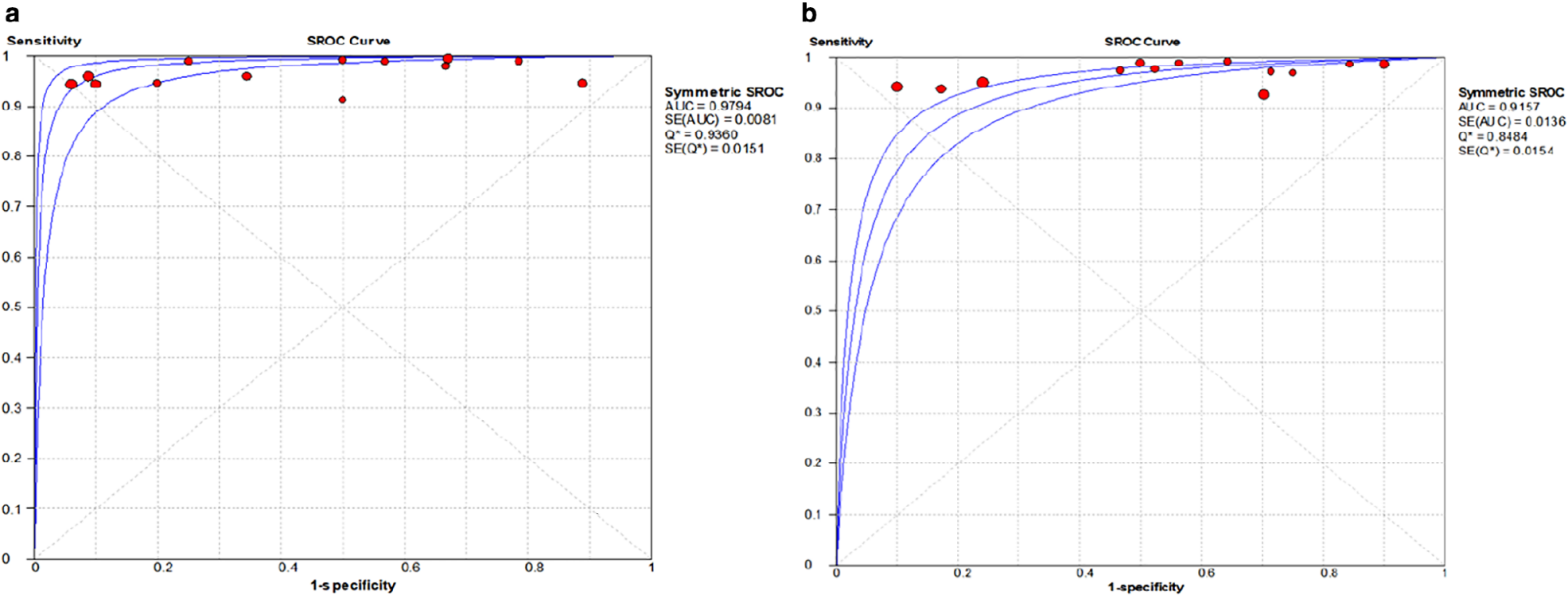
The ROC curve of CESM and MRI for breast cancer diagnosis. (**a)** ROC curve for CESM in the diagnosis of breast cancer; (**b**) ROC curve for MRI in the diagnosis of breast cancer.

The combined data indicated the pooled sensitivity (Fig 3) and specificity (Fig 4) of CESM and MRI were 0.97 (95% CI: 0.95–0.98), 0.66 (95% CI: 0.59–0.71) and 0.97 (95% CI: 0.95–0.98), 0.52 (95% CI: 0.46–0.58), respectively. The pooled +LR (Fig 5) and –LR (Fig 6) were 2.70 (95% CI: 1.57–4.65), 0.06 (95% CI: 0.04–0.09) for CESM and 2.01 (95% CI: 1.78–2.26), 0.08 (95% CI: 0.05–0.11) for MRI, respectively. For the diagnostic odds ratio (DOR), the pooled results of CESM and MRI were 60.15 (95% CI: 24.72–146.37) and 31.34 (95% CI: 19.61–50.08), respectively (Fig 7). The AUC of the symmetric receiver operating characteristic curve (SROC) was 0.9794 and 0.9157 for CESM and MRI, respectively, calculated through the Moses model for, or in the diagnosis of, breast cancer (Fig 8). The summary of the pooled diagnostic performance of CESM and MRI was demonstrated in Table 2.

**Table 2:** The summary of the pooled diagnostic performance of CEMS and MRI in breast cancer diagnosis

Diagnostic parameter	CESM	MRI
Sensitivity	0.97 (95% CI: 0.95–0.98)	0.97 (95% CI: 0.95–0.98)
Specificity	0.66 (95% CI: 0.59–0.71)	0.52 (95% CI: 0.46–0.58)
+LR	2.70 (95% CI: 1.57–4.65)	2.01 (95% CI: 1.78–2.26)
–LR	0.06 (95% CI: 0.04–0.09)	0.08 (95% CI: 0.05–0.11)
DOR	60.15 (95% CI: 24.72–146.37)	31.34 (95% CI: 1961–50.08)
AUC	0.9794	0.9157

## Discussion

In this meta‐analysis, we included 13 studies which compared the diagnostic performance of CESM and MRI used in the detection of breast cancer. The general quality evaluation analysis of the 13 publications indicated low risk of bias patient selection and reference standard, but high risk of flow and timing. Therefore, the general quality of the included studies was moderate, which indicated that the flow and timing aspect should be improved in future relevant clinical studies. The combined results showed that the diagnostic sensitivity of CESM and MRI was both high without significant difference. The high diagnostic sensitivity indicated both CESM and MRI are effective methods for breast cancer screening. However, the diagnostic specificity of CESM and MRI were relatively low (0.66 and 0.52). The low specificity induced high false positive diagnostic results which may lead to excessive unnecessary and invasive diagnostic procedures such as needle biopsy. Breast cancer diagnosis by either CESM or MRI should be further confirmed by other diagnostic methods. The AUC of the symmetric receiver operating characteristic curve (SROC) was 0.9794 and 0.9157 for CESM and MRI, respectively calculated through the Moses model for, or in the diagnosis of, breast cancer. AUC for CESM and MRI were both very high and close to “1.0”, indicating the diagnostic performance of CESM and MRI were good. Both CESM and MRI are effective methods for detection breast cancer with high diagnostic sensitivity. However, the diagnostic performance of CESM seems to be more effective than MRI in the detection of breast cancer according to the AUC.

Routine mammography is an easy and convenient procedure, but its sensitivity is low due to radiologically dense epithelial and connective breast tissue including the glands, especially in oriental women.^18^, ^19^ Misdiagnosis frequently occurs due to the overlapping pathological appearance. Ultrasound examination, another method used to identify breast cancer, is radiation‐free, but highly dependent on the experience of the operator, with a high false‐positive rate of breast lesion diagnosis.^20^ MRI in the diagnois of breast cancer has high sensitivity but low specificity. However, MRI examination is time‐consuming and expensive, and is not the first treatment of choice in breast cancer screening or diagnosis.^21^, ^22^ CESM is a new technique based on traditional mammography and intravenous iodine contrast technology. The metabolism of cancer cells differs markedly from that of healthy cells, as cancer cells secrete growth factors that promote the formation of new blood vessels during division and proliferate into tumor cells. The blood vessels have increased endothelial space and permeability, which enhance the contrast in the tumor area. CESM can improve the accuracy of detection and differentiation of breast lesions by providing information about the degree of neovascularization.

In conclusion, CESM is reported to have a higher degree of accuracy in the diagnosis of breast cancer. Compared with MRI, it has a higher specificity and diagnostic performance, and can be used as an effective tool in the screening and diagnosis of breast cancer.

## Disclosure

The authors declare there are no conflicts of interest.
